# Effects of Pyruvate Administration on Mitochondrial Enzymes, Neurological Behaviors, and Neurodegeneration after Traumatic Brain Injury

**DOI:** 10.14336/AD.2020.1015

**Published:** 2021-07-01

**Authors:** Prasanth S Ariyannur, Guoqiang Xing, Erin S Barry, Brandi Benford, Neil E Grunberg, Pushpa Sharma

**Affiliations:** ^1^Department of Anesthesiology, Uniformed Services University of the Health Sciences, Bethesda, MD 20814, USA.; ^2^Imaging Institute of Rehabilitation and Development of Brain Function, the Affiliated Hospital and the Second Clinical Medical College of North Sichuan Medical University, Nanchong Central Hospital, Nanchong 637000, China.; ^3^Department of Biochemistry & Molecular Biology, Amrita Institute of Medical Sciences, Amrita Vishwa Vidyapeetham, Kochi 682041, India.; ^4^Military & Emergency Medicine, Uniformed Services University of the Health Sciences, Bethesda, MD 20814, USA.

**Keywords:** Fluid percussion, mTBI, mitochondrial enzyme, neurodegeneration, pyruvate

## Abstract

Traumatic brain injury (TBI) is known to increase the susceptibility to various age-related neurodegenerative disorders such as Alzheimer’s disease (AD) and Parkinson’s disease (PD). Although the role of damaged mitochondrial electron transport chain (ETC) in the progression of AD and PD has been identified, its relationship with altered expression of neurodegenerative proteins has not been examined before. This study aimed to investigate 1) how TBI could affect mitochondrial ETC and neurodegeneration in rat brain regions related to behavioral alteration, and 2) if administration of the key mitochondrial substrate pyruvate can improve the outcome of mild TBI (mTBI). In a rat lateral fluid percussion injury model of mTBI, sodium pyruvate in sterile distilled water (1 g/kg body weight) was administered orally daily for 7 days. The protein expression of mitochondrial ETC enzymes, and neurodegeneration proteins in the hippocampus and cerebral cortex and was assessed on Day 7. The hippocampal and cortical expressions of ETC complex I, III, IV, V were significantly and variably impaired following mTBI. Pyruvate treatment altered ETC complex expression, reduced the nitrosyl stress and the MBP expression in the injured brain area, but increased the expression of the glial fibrillary acidic protein (GFAP) and Tau proteins. Pyruvate after mTBI augmented the Rotarod performance but decreased the horizontal and vertical open field locomotion activities and worsened neurobehavioural severity score, indicating a debilitating therapeutic effect on the acute phase of mTBI. These results suggest bidirectional neuroprotective and neurodegenerative modulating effects of pyruvate on TBI-induced alteration in mitochondrial activity and motor behavior. Pyruvate could potentially stimulate the proliferation of astrogliosis, and lactate acidosis, and caution should be exercised when used as a therapy in the acute phase of mTBI. More effective interventions targeted at multiple mechanisms are needed for the prevention and treatment of TBI-induced long-term neurodegeneration.

Traumatic brain injuries (TBI) and neurodegenerative diseases are thought to be two independent causes of death and disabilities [1]. Over the past 30 years, however, studies have linked TBI as the potential risk factors of Alzheimer’s disease (AD) and dementia [2, 3]. Neuropathological studies of TBI patients have shown the accumulation of amyloid plaques following a single severe TBI, and Tau pathology after repeated moderate (mTBI), which are indicators of AD development [3, 4]. Based on these and other observations, the blast-related moderate to severe TBI may also increase the risk of developing dementia and AD later in life [5, 6]. So far, the underlying mechanisms and effective treatment for TBI-induced neurodegenerative diseases have not been fully explored.

The impaired mitochondrial function would result in depleted cellular ATP production, the energy source of neuronal activity and brain function, and increased production of reactive oxygen species, a major cause of neural cell death and neurodegeneration [7]. Mitochondria generate cellular ATP by metabolizing glucose-derived pyruvate through the mitochondrial pyruvate dehydrogenase complex (PDH) and oxidative phosphorylation through the electron transport chain (ETC). The ETC is composed of five multi-subunit enzyme complexes: NADH-ubiquinone reductase (complex I), succinate-ubiquinone reductase (complex II), ubiquinone-cytochrome c reductase (complex III), cytochrome c oxidase (complex IV), and ATP synthase (complex V). A disruption in the mitochondrial ATP producing pathway would result in increased oxidative damage to the cellular protein, lipid, and nuclear components in the brain leading to neurodegenerative pathologies [8]. Despite the progress in the experimental pathophysiology of mTBI, limited success has been achieved in finding effective therapies for mTBI patients. We hypothesize that agents than can improve/protect mitochondrial metabolism/integrity may prevent TBI-induced neurodegeneration.

Exogenous pyruvate represents a novel avenue for the prevention and treatment of TBI-related cognitive deficits and neurodegenerative disorders, providing metabolic support to the mitochondria after TBI. Pyruvate is the product of glycolysis, a substrate, and a natural allosteric activator of the PDH enzyme. Pyruvate can easily cross the blood-brain barrier, improve neuronal energy supply via the TCA cycle, intracellular calcium buffering, reduced apoptosis, and increased cognitive behavior [9-13]. In this study, we examined the biomarkers of brain mitochondrial activity (ETC), neurodegeneration (Glial Fibrillary Acidic Protein, GFAP), Tau, Myelin Basic Protein (MBP) and reactive nitrogen species (Nitro-Tyrosine (N-Tyr), and behavioral outcomes in a rat model of mTBI with and without pyruvate treatment.

## MATERIALS AND METHODS

### Animals and treatment groups

Adult male Sprague-Dawley rats (225-275g) were purchased from Harlan Laboratories (Indianapolis, IN), and paired housed in standard cages in a temperature-controlled facility with 12-h reversed light-dark cycle. All procedures were performed per the Office of Laboratory Animal Welfare and NIH guidelines, and the protocol was approved by the Institutional Animal Care and Use Committee (IACUC) of Uniformed Services University of the Health Sciences. Animals were given food and water ad libitum. Rats were given coded tail numbers and assigned randomly to each of the four experimental groups. Mild TBI (mTBI) was induced by lateral fluid percussion injury according to our published procedure [10]. Group 1: naïve + vehicle (n=8). Group 2: naïve + pyruvate (n=8). Group 3: mTBI + vehicle (n=12): Group 4: mTBI +pyruvate (n=12). Naïve animals were time matched with vehicle treatment (sterile water or pyruvate of the same volume) that did not receive any brain injury. Animals in pyruvate treatment groups received sodium pyruvate (1g/kg in sterile distilled water) orally every 24 h for seven days. Baseline behavioral tests were conducted and were tested again during the first seven days post-TBI.

### Fluid percussion injury and tissue collection

Rats were anesthetized with 1-3% isoflurane in oxygen. Under sterile conditions, a 3 cm sagittal incision was made along the midline to expose the cranium. A 5 mm burr hole was created 2 mm to the right of the sagittal suture halfway between bregma and lambda using a 5 mm trephine drill bit exposing the dura. A Luer-Lock needle hub was placed into the burr hole and cemented to the cranium using cyanoacrylate glue. The glue was allowed to completely dry, and the empty Luer-lock hub was filled with normal saline before being connected to the TBI device. mTBI was induced by a fluid percussion pulse of 2.0 - 2.5 atm administered by an injury cannula positioned parasagittally over the right cerebral cortex [14]. The fluid percussion pulse was administered by a pendulum modulated fluid percussion biomechanical device (Richmond, VA, USA). The Luer-lock hub was removed and defects in the cranium were repaired with bone wax. The skin was closed with a surgical skin stapler and animals were allowed to stabilize in the warm blanket before returned to their home cages. At 7-day post-injury, animals were sacrificed under isoflurane anesthesia. Brains were removed followed by the dissection of the ipsilateral and contralateral hippocampus and cerebral cortex of mTBI. Three animals from each group were perfused with neutral buffered formalin for brain immunohistochemistry.

### Animal behavior and functional measures

Animal behavior was observed during its dark cycle. Behaviors were measured before the injury (baseline), and at various periods following TBI with pyruvate or an equal volume of distilled water as a vehicle. Behavioral measures included: open-field activity (OFA) to measure general health, depression- and anxiety-related behaviors; neurobehavioral testing (NSS-R) to measure sensory-motor functioning; rotarod (RR) to measure motor performance due to neuromuscular impairment.

### Open field activity

OFA measures naturally occurring behaviors that are exhibited when an animal explores and interacts with its surroundings. These measures provide data about gross motor movement and specific movements related to psychological conditions (e.g., anxiety-like and depressive-like behaviors [15]. In this experiment, two variables were identified from the animal’s movement within the chambers: horizontal activity (HA, an index of general health and gross motor movement) and vertical activity (VA, less VA is considered to be less escape and more depressive-like behavior). OFA was measured according to the method described by Grunberg and Bowen [16] using Accuscan Superflex Sensor Version 2.2 infrared photocell system in the Accuscan Instruments testing chamber (measuring 40 x 40 x 30 cm; Accuscan Instruments Incorporated, Columbus, OH) located in a dedicated room designed to minimize acoustic interruptions. Animals were acclimated to the chambers before the beginning of the experiment. They then received a baseline measurement before the injury and were measured at days 3 and 5 post-injury. The open field activity of each rat was measured for 1 hr. during its active period (dark cycle).

### Revised Neurobehavioral Severity Scale (NSS-R)

The NSS-R is a sensitive and reliable measure of sensory-motor responses in rodents [17]. This measure models a clinical neurological exam of human patients and was based on several previous reports [18] and has been modified to increase standardization. It is a specific, continuous sequence of behavioral tests and observations [17]. The tests assess reflex suppression, general movement, and postural adjustments in response to a challenge. The NSS-R uses a three-point Likert scale, in which a normal, healthy response is assigned a “0”, a partial or compromised response is assigned a “1”, and the absence of a response is assigned a “2”. This three-point scale is clear and reliable and allows for greater discrimination based on sensory-motor responses than do previous scales that used two-point ratings of each response. The NSS-R has a scoring range of 0-20 with higher scores reflecting a greater extent of injury severity. Three NSS-R sessions were conducted 3 times in this experiment: one before the injury (baseline) and two after injury (days 3 and 5 post mTBI).

### Rotarod Activity

The rotarod measures the animal’s motor coordination. Motor coordination in rodents reflects balance, muscle strength, and fatigue [19]. A Four Station Rotarod Standalone for rat machine (ENV-577, Med Associates Inc, Georgia, Vermont), using a procedure based on previous reports [19, 20]. Briefly, the rats were placed on the stationary rod facing the wall. Once the trial was initiated, rotation speed began at 4 rpm and increased gradually to 40 rpm. The latency until the maximum speed was 300 sec. Rats falling from the rod, a height of 26.7 cm, would break the photo beam stopping the timer. The duration, the rat-maintained its position on the rod was recorded in seconds, with a maximum of 300 sec. All rats were tested before the injury (baseline), and post-injury (4- and 6- days post mTBI). The rats underwent three consecutive trials at each testing and the average value (in seconds) was used for analyses.

### Mitochondrial ETC enzymes in hippocampus and cortex by Western blotting

Brain tissues of the contralateral and ipsilateral sides of the hippocampus, and prefrontal cortex (PFC) were homogenized and sonicated in the T-Per tissue lysis buffer (Pierce, IL, USA). Protein concentrations were determined using a Bradford assay (BioRad, CA, USA). Aliquots of 20 µg proteins were separated by electrophoresis on the NuPage Novex Midi Bis-Tris gels (4-12%) (Invitrogen, CA) and transferred to a polyvinylidene difluoride membrane (Millipore, USA). The membranes were rinsed in a 0.01 M Tris-buffered saline solution (pH 7.4, 0.1% Triton X-100) for 30 minutes, blocked in 5% bovine serum albumin for another 30 minutes, and incubated overnight at 4°C with the primary mouse monoclonal antibodies for the mitochondria control protein citrate synthase and the ETC complexes I-V (Total OXPHOS Rodent WB Antibody Cocktail, Abcam, USA) with 1:200 dilution each in a Tris-buffered saline solution containing 3% bovine serum albumin. The membranes were washed three times with Tris-buffered saline solution for 30 min and incubated at room temperature with a horseradish peroxidase-conjugated secondary anti-mouse antibody (1:5,000 dilution) in the Tris-buffered saline solution for 60 min. Immunoreactive bands were visualized using ECL Western blotting detection reagents (GE Healthcare Bio-Sciences Corp, Piscataway, NJ). The western blots were captured with a charged coupled device camera (CCD camera) and the intensities of the specific protein bands were quantified with NIH Image software.

### Immunohistochemical analysis

The effect of injury as well as the reactive and neuronal degenerative changes in the cortical regions of injury were assessed by immunohistochemical analysis. GFAP immunoreactivity was assessed using a rat anti-GFAP antibody (Thermo Fisher Scientific, CA) for post-injury glial cell proliferation. For the evaluation of post-injury neurodegeneration, immunoreactivities of MBP (using rabbit anti-MBP antibody), and Tau protein (using mouse monoclonal anti-Tau antibody), both antibodies were obtained from Millipore, Sigma USA. The neuronal injury by oxidative damage was determined using Nitro-tyrosine (mouse anti-N-tyrosine antibody from Abcam). Immunoreactivity of all these markers was evaluated in cortical pericontusional regions, hippocampal dentate gyrus regions in the brain sections of rats from naïve + vehicle, mTBI + vehicle, and mTBI+ pyruvate groups.

Fluorescence Immunohistochemistry was performed according to the method described earlier [21] with minor modifications. Under isoflurane anesthesia, intra-cardiac perfusion of buffered formalin (pH 7.4) was performed in three rats from each group. The perfusion was assessed using the level of blanching of extremities and muscle rigidity. After decapitation, the brain was carefully dissected out and stored in neutral buffered formalin at room temperature. After 24 hours, the brain was transferred into a serial ascending concentration of sucrose, 10%, 20%, and 30% in PBS, to cryopreserve the tissue. The brains were, embedded in the Tissue Tek^®^ cryo-embedding OCT compound (Sakura Finetek Japan Co. Ltd), sectioned on a cryostat at -20?, 20 µm thickness per section. The sections were directly mounted on super frost charged glass slides and preserved in -80°C until further use. Rabbit Anti-MBP antibody was diluted to 1:1000 and all other antibodies were diluted to 1:100 with 1% normal goat serum in PBS with 0.3% Triton-X 100. Tissue sections on glass slides were incubated with diluted antibodies overnight at room temperature. After removal of primary antibodies, slides were washed with PBS containing 0.2% Triton X-100 for three times. An appropriate secondary antibody was subsequently incubated with the sections for one hour at room temperature. Secondary antibodies raised in Goat (Goat Anti-mouse IgG or Goat anti-Rat IgG or Goat anti-Rabbit IgG) coupled to AlexaFluor 488 (Thermo Fisher Scientific) for green fluorescence, and coupled to AlexaFluor 594 (Thermo Fisher Scientific) for red fluorescence were used appropriately to provide contrasting color for colocalization studies. Cell nuclei were stained blue using DAPI, just added before coverslip. The images were captured using a Zeiss-PASCAL LSCM and were edited using Zeiss Image Examiner version 5.0 (Macintosh version).

**Figure 1.**
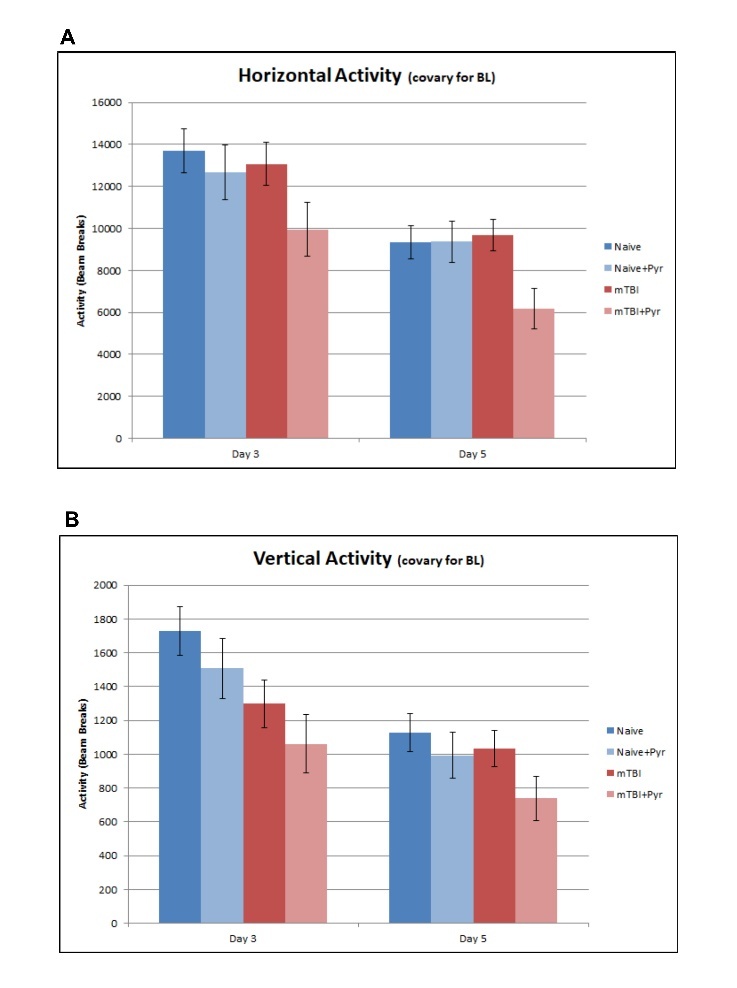
Open field activity. (A) Horizontal activity at day 3 and 5 post-TBI or naïve animals with or without pyruvate treatment. Horizontal activity is an index of the general health and movement of the animals. There were significant differences at baseline therefore baseline measurements were used as a covariate for further analyses. (B) Vertical activity at day 3 and 5 post-TBI or naïve animals with or without pyruvate treatment. Vertical activity is an index of depression-related behaviors where a decrease in activity indicates more depression-related behavior. There were significant differences at baseline therefore baseline measurements were used as a covariate for further analyses.

### Statistical analysis

For behavioral data analyses, analyses of variance (ANOVA; covarying for baseline measurements where needed) and repeated measures analysis of variance (rANOVA; covarying for baseline measurements where needed) were conducted for each of the dependent variables. Dunnett’s t-tests were performed where appropriate to compare all groups to the naïve animals. Open field activity scores were separated into two subscales: horizontal activity and vertical activity. Analyses for all measures except for OFA included data for all subjects (N=48). The open field activity included a subset of the animals (N=40) due to an equipment malfunction. Cohorts were similar among experimental groups; therefore, the remaining data are representative of all experimental conditions. All tests were two-tailed using alpha=0.05. Data analyses were performed at the end of the experiment after all measurements were collected. For mitochondrial Western blot analysis of protein band intensities, one-way ANOVA for multiple comparisons was used, and p < 0.05 was considered significant.

**Figure 2.**
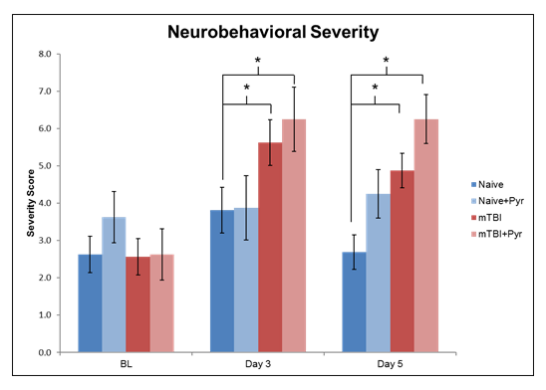
The revised neurobehavioral severity scale at baseline, day 3, and day 5 post-TBI or naïve animals with or without pyruvate treatment. The revised neurobehavioral severity scale is 10 consecutive tasks designed to measure sensory and motor reflexes scored from 0-2; where 0 is a completely normal response, 1 is a partial response, and 2 is no response.

## RESULTS

### Behavioral and functional outcomes of mTBI and pyruvate treatment

#### Open field activity (OFA)

*Horizontal Activity-* The HA of the animals is shown in Figure 1A. Less HA indicates fatigue, general health, and movement of the animals. Because of the baseline differences in horizontal activity (HA), baseline measurements were used as a covariate for HA analyses (Fig. 1A). Overall, there is a progressive decline in HA from day 3 to day 5 post in both naïve control and mTBI groups, probably due to increased familiarity with the activity chamber. Pyruvate treatment significantly reduced HA [F(1,34) = 4.23, p = 0.047, η2 = 0.11] compared to non-pyruvate treated animals. A significant mTBI+ pyruvate interaction, F(1,34) = 4.23, p = 0.048, η2 = 0.11 indicate a significant HA reduction in the mTBI + pyruvate group but not in the naïve + pyruvate group. Pairwise comparisons revealed that the mTBI + pyruvate animals (8061.8 ± 1046.7) had significantly less HA than the naïve animals (11521.7 ± 863.3; p= 0.017) and the mTBI animals (11369.6 ± 832.2; p = 0.016). mTBI + pyruvate animals had significantly lower amounts of HA (9951.01 ± 1285.2) than naïve animals (13695.7 ± 1059.9; p = 0.034) at day 3 post-injury. There was a main Group effect at day 5 post-injury, F(3,34) = 3.31, p = 0.032, η2 = 0.23, such that mTBI + pyruvate animals (6172.7 ± 47.6) had significantly less HA than did naïve animals (9347.8 ± 781.5; p = 0.016), the naïve + pyruvate animals (9361.83 ± 965.16; p = 0.031), and the mTBI animals (9677.79 ± 753.37; p = 0.005). Similarly, there was a main effect of pyruvate treatment, F(1,34) = 4.34, p = 0.045, η2 = 0.11, such that pyruvate-treated animals (7767.24 ± 642.93) had significantly less HA than animals that did not receive pyruvate (9512.79 ± 536.42).

*Vertical Activity-* The VA of the animals is shown in Figure 1B. Less vertical activity indicates depression-related behaviors. Because VA differed at baseline among groups, baseline measurements were used as a covariate in subsequent analyses. Overall, pairwise comparisons revealed that mTBI + pyruvate animals (900.0 ± 145.7) had significantly less VA than did naïve animals (1427.9 ± 122.8; p = 0.009). There was a main effect for injury, F(1,34) = 4.74, p = 0.037, η2 = 0.12, such that injured animals (1033.1 ± 95.3) had significantly less VA than did non-injured animals (1338.3 ± 97.8). There also was a Time × Group interaction, F(3,34) = 3.6, p = 0.022, η2 = 0.24, and a Time × Injury interaction, F(1,34) = 8.6, p = 0.006, η2 = 0.20. At day 3 post injury, there was a main effect for Group, F(3,34) = 3.2, p = 0.04, η2 = 0.22, such that naïve animals (1727.9 ± 144.5) had significantly more VA than did mTBI animals (1298.1 ± 140.1; p = 0.041) and mTBI + pyruvate animals (1061.3 ± 171.5; p = 0.006). There also was a main effect for injury, F(1,34) = 7.00, p = 0.012, η2 = 0.17, such that injured animals (1179.7 ± 112.2) had significantly less VA than did non-injured animals (1617.4 ± 115.1). At day 5 post injury, there were no main effects, but pairwise comparisons revealed that naïve animals (1128.1 ± 111.1) had significantly more VA than did mTBI + pyruvate animals (738.8 ± 131.9; p = 0.031).

#### Revised neurobehavioral severity scale (NSS-R)

Figure 2 presents the neurobehavioral severity data (NSS-R; higher scores indicate more sensory-motor functional impairment) of the animals. All groups were comparable at baseline. Overall, there was a main effect for Time, F(2,88) = 14.1, p < 0.001, η2 = 0.24, such that baseline scores (2.9 ± 0.30) were significantly less than day 3 (4.9 ± 0.4; p < 0.001) and day 5 post injury (4.5 ± 0.3; p < .001) scores. At day 3 post injury, the differences between groups approached significance (p = 0.051), where pairwise comparisons revealed that naïve animals (3.8 ± 0.61) scored significantly lower than did mTBI animals (5.7 ± 0.61; p = 0.042) and mTBI + pyruvate animals (6.3 ± 0.86; p = 0.026). At day 5 post injury, there was a main effect for Group, F(3,44) = 7.55, p < 0.001, η2 = 0.34, such that naïve animals (2.69 ± 0.46) scored significantly lower than did mTBI animals (5.6 ± 0.611; p = 0.002; Dunnett’s t, p = 0.005) and mTBI + pyruvate animals (6.3 ± 0.86; p < 0.001; Dunnett’s t, p < 0.001). Naïve + pyruvate animals (4.3 ± 0.65) had significantly lower scores than did mTBI + pyruvate animals ( p = 0.036). There was a main effect for Injury, F(1,44) = 13.70, p = 0.001, η2 = 0.24, such that injured animals (5.6 ± 0.40) had significantly higher scores than did non-injured animals (3.5 ± 0.40). There was also a main effect for pyruvate, F(1,44) = 6.74, p = 0.013, η2 = 0.13, such that pyruvate-treated TBI animals (5.3 ± 0.46) had significantly higher score than did TBI animals that did not receive pyruvate (3.8 ± 0.33).

**Figure 3.**
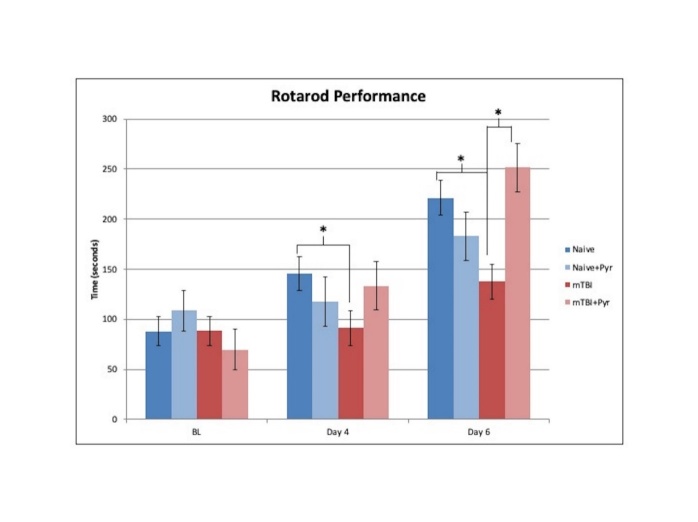
Rotarod performance at baseline, and day 4 and 6 in rats with mTBI or naïve treated with/out pyruvate. mTBI animals had significantly worse performance than did naïve animals at days 4 and 6. At 6 days, post-injury mTBI animals had significantly worse performance when compared with naïve animals. Pyruvate significantly increased the rotarod performance in mTBI groups compared with naïve animals.

**Figure 4.**
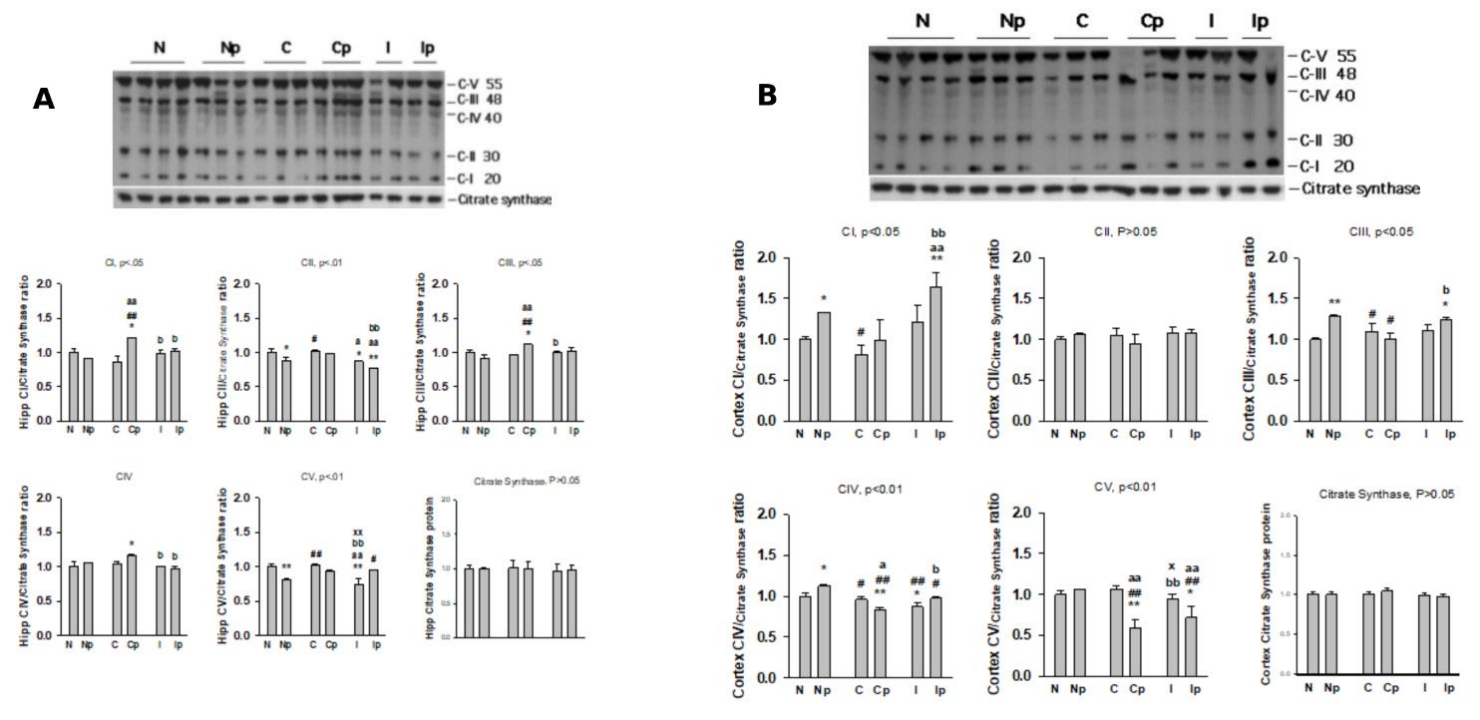
ETC Enzymes in Hippocampus and Cortex in response to mTBI and pyruvate treatment. The Hippocampus (A) and cortex (B) from the injured and uninjured areas of the rat brain were collected at day 7 from post-injury or naïve animals. Expression of mitochondrial electron transport chain complexes I-V and mitochondria house-keeping citrate synthase were determined by Western blotting. Typical western blot images are presented on the top and the results are expressed as the integrated density of protein bands (mean ±S.D.). N, Naive control group; P, Naïve control group plus1 M Pyruvate treatment; C: contralateral cortex/hippocampus of TBI group; Cp: contralateral cortex/hippocampus of TBI plus 1M pyruvate treatment; I: ipsilateral cortex/hippocampus of TBI; Ip: ipsilateral cortex/hippocampus of TBI plus 1M pyruvate treatment. * p<0.05, ** p<0.01 vs. naïve control; #<0.05, ## p<0.01 vs. naïve control + pyruvate; a<0.05, aa p<0.01 vs. contralateral site of TBI; b<0.05, bb <0.01 vs. ipsilateral site of TBI; x<0.05, xx <0.01 vs. ipsilateral site of TBI + pyruvate.

#### Rotarod

Figure 3 presents rotarod performance data. There were no significant differences among groups at baseline. Overall, there was a main effect for time such that baseline (88.6 ± 8.8) had significantly lower times than day 4 post injury (122.1 ± 10.5; p= 0.007), and day 6 post injury (198.4 ± 10.6; p<0.001). Day 4 post injury had significantly less time on the rotarod than at day 6 post injury (p<0.001). There was a main effect for group, F(3,44) = 3.22, p = 0.032, η2 = 0.18, such that mTBI animals (105.8 ± 11.5) had significantly lower performance times than did naïve animals (151.7 ± 11.45; p= 0.007; Dunnett’s t, p= 0.019), and mTBI + pyruvate animals (151.5 ± 16.2; p= 0.026). There was also a significant injury × pyruvate interaction, F(1,44) = 4.8, p = 0.035, η2 = 0.10, Time × Group interaction, F(6,88) = 3.11, p = 0.008, η2 = 0.18, and time × injury × pyruvate interaction, F(2,88) = 7.6, p = 0.001, η2 = 0.15. At 3 days post injury revealed that mTBI animals (91.3 ± 17.3) had significantly worse performance than did naïve animals (145.9 ± 17.2; p = 0.029). At 5 days post injury, mTBI animals (137.8 ± 17.3) had significantly worse performance when compared with naïve animals (221.2 ± 17.3; p = 0.001; Dunnett’s t, p = 0.004) and mTBI + pyruvate animals (251.5 ± 24.4; p < 0.001).

### Pyruvate prevents mitochondrial ETC enzymes degradation in the brain hippocampus and cortex after mTBI

#### Hippocampus

Data presented in Figure 4A depicts that compared with naive animals, mTBI with/out pyruvate treatment had significant effects on the expression of CI, CII, CIII, CV (p < 0.01), and a trend effect on CIV (p < 0.15) protein expression in rat hippocampus. The expression of CI, CIII, and CIV, but CII, CV, and PDHE1α were significantly decreased in the ipsilateral hippocampus (injured side) compared with contralateral (uninjured side). Pyruvate treatment significantly increased CI, CIII, in the contralateral hippocampus, but in the ipsilateral side, CV was increased and PDHE1α was decreased. This indicates a selective role of pyruvate in preventing the loss of mitochondrial ETC in the hippocampus at day 7 following TBI.

#### Cerebral cortex

Compared with naïve animals, mTBI with/out pyruvate treatment had significant effects on the expression of ETC components CI, CIII, CIV, CV, and PDHE1α (p < 0.01) protein levels in the ipsilateral cerebral cortex (Fig. 4B). Compared with naïve animals, the contralateral cortex (uninjured site) had significantly higher CI, while CIV was significantly lower than the ipsilateral cortex (injured site). Pyruvate administration significantly increased CI, CIII, CIV, but decreased CV in the ipsilateral side compared with that of naïve animals and contralateral cortex (no direct injury), indicating a protective effect of pyruvate in the cerebral cortex of mTBI animals.

**Figure 5.**
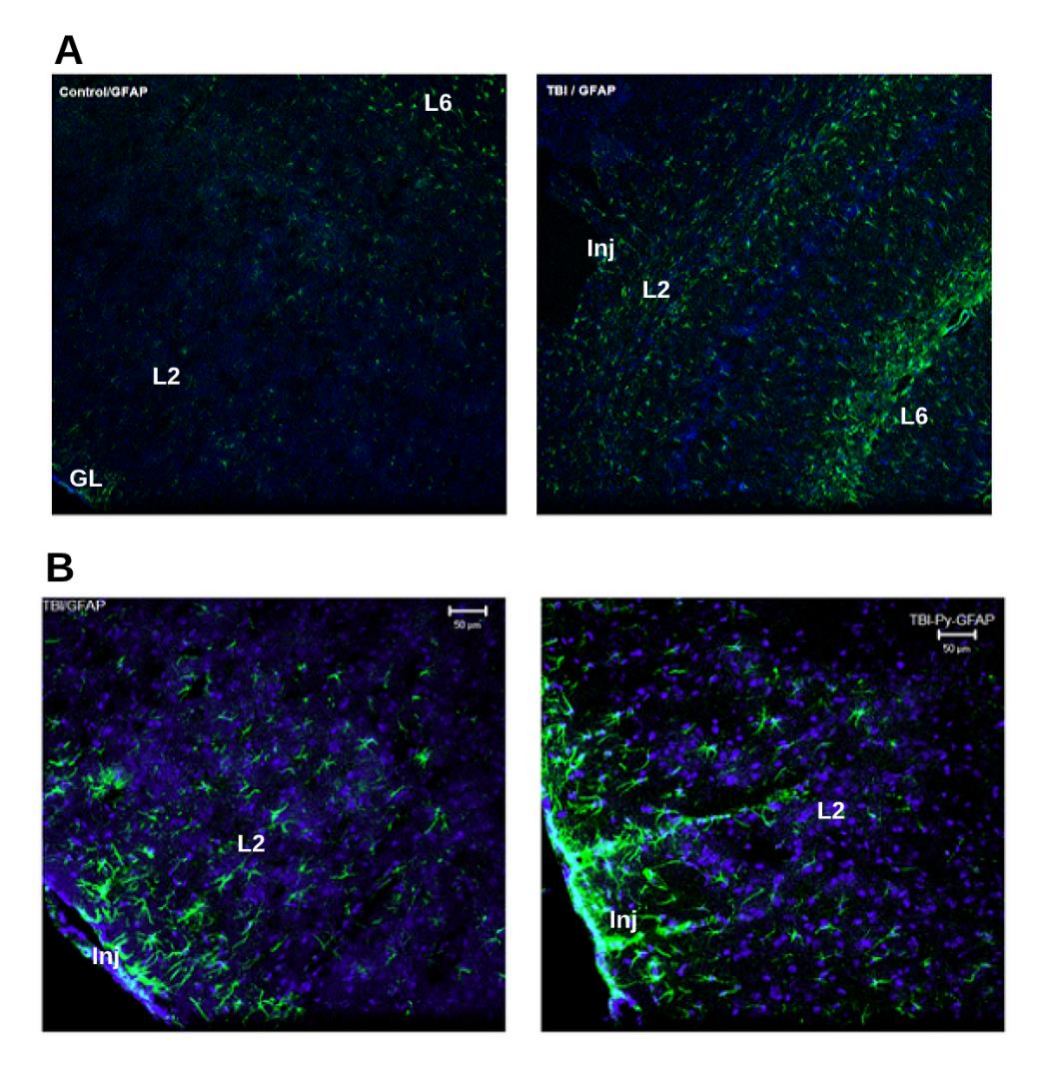
GFAP Immunolocalization in rat brain cortex with/out injury. Green fluorescence represents immunolocalization of GFAP and nuclei is stained blue by DAPI. (A) Image on the left-side (marked “Control/GFAP”) is typical appearance of a rat cerebral cortical region without injury (naïve+vehicle group). Image on the right-side (marked “TBI/GFAP”) is from the brain of a rat seven days after TBI (mTBI + vehicle group). Images are acquired on a 10x obj. on Zeiss LSCM. Similar iso-cortical regions of different rat brain sections are compared. Region marked “GL” is glial limitans seen in the uninjured brain. Regions marked “L2” and “L6” are isocortical pyramidal layers. Region marked “Inj” is the injured area of cortex. The “GL” is split apart from the cortical layers in the “Inj” region in mTBI sections. GFAP expression is more with white matter than grey matter. There is an overall increase in GFAP expression in the cortex of mTBI rats compared to that of naïve. (B) GFAP Immunolocalization in post TBI rat brain cortex with / out pyruvate treatment. Left side image (marked “TBI/GFAP”) are from mTBI + vehicle group and image on the right-side (marked “TBI-Py-GFAP”) are from mTBI+pyruvate group. Images were obtained on a 20x obj. There is an overall increase in GFAP expression in pyruvate treated rat brain than non-treated brain post-injury.

### Immunohistochemistry

#### GFAP expression in TBI

As a major component of astroglia, GFAP immune-histochemistry was used to visualize astroglial changes in the hippocampus and cortex. There have been numerous studies in TBI sub-population such as severe or moderate TBI where GFAP concentration has shown a positive correlation with severity of injury [22]. Figure 5 shows the typical immunoreactivity (IR) of GFAP in the cortical brain regions of animals from different cohorts. The immunoreactivity of GFAP in the perilesional injured cortical brain region of animals in the mTBI group is higher than that of an uninjured cortical region, showing the occurrence of reactive gliosis after seven days of injury (Fig. 5A). But the expression of GFAP was higher in cortical and sub-cortical regions of brains of pyruvate treated TBI (mTBI + pyruvate) compared to the untreated group (mTBI, Fig. 5B). This shows that generally, the pyruvate treatment increases GFAP IR in perilesional isocortical regions.

**Figure 6.**
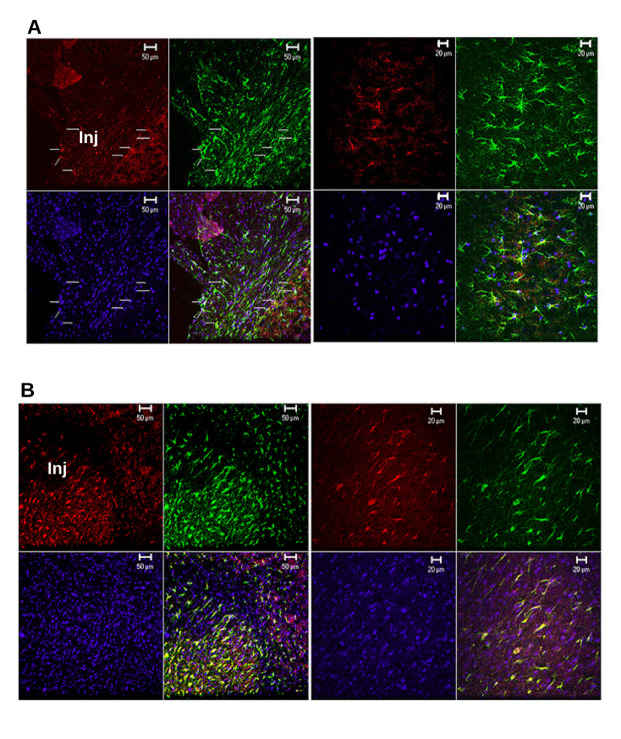
Immunohistochemical colocalization of GFAP and Tau in post-TBI untreated and pyruvate treated rat frontal brain. (A) Colocalization of GFAP and Tau in frontal brain sections of post- mTBI rats not treated with sodium pyruvate (mTBI + vehicle group). Green fluorescence for GFAP and red for Tau protein and cell nuclei were stained blue using DAPI. On the left side shows a typical immunolocalization of perilesional cortex (20x obj.). Region marked “Inj” is the cortical region of impact of mTBI. Arrows show co-localization for Green and Red as Yellow fluorescence. The image on the right-side shows a typical immunolocalization seen in hippocampal CA1 and Dentate Gyrus. (B) Colocalization of GFAP and Tau in frontal brain sections of post-TBI rats treated with sodium pyruvate (mTBI+pyruvate group). The left side image is from the perilesional cortex (20x obj.). Region marked “Inj” is the cortical region of impact of mTBI. The image on the right-side is acquired from the CA1 hippocampus (40x obj). Compared to Fig 6A, the intensity of immunoreactivity of both GFAP and Tau is higher in sections of brains from mTBI+pyruvate group with a larger number of immunoreactive cells. This suggests that there is a higher amount of expression of both GFAP and Tau expression in post-TBI sodium pyruvate treatment compared to untreated TBI controls.

**Figure 7.**
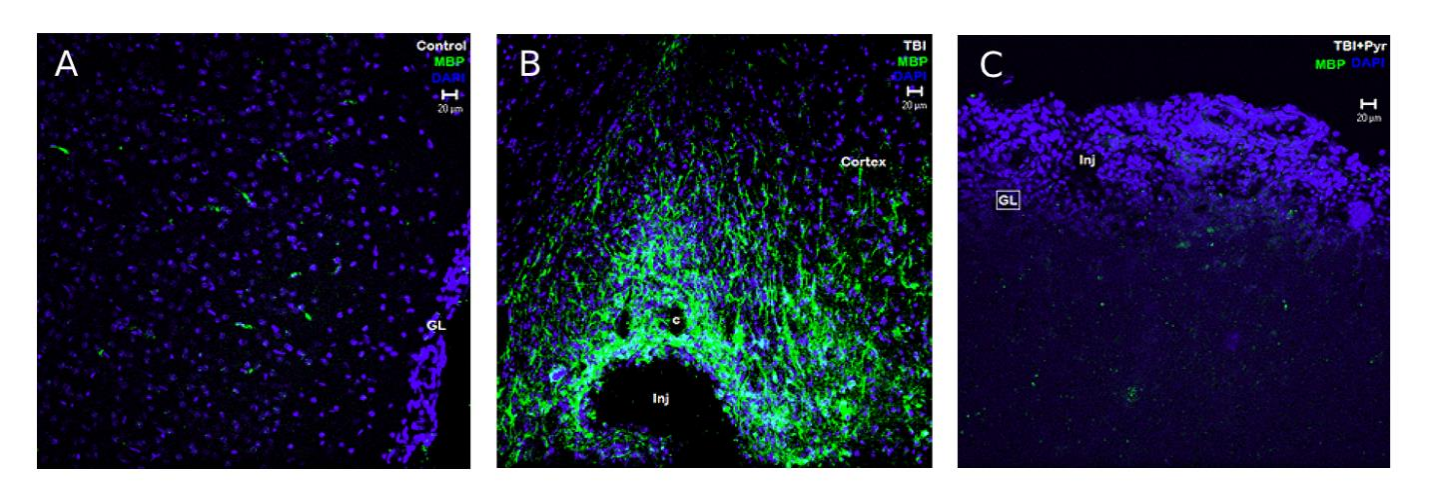
Immuno-colocalization of MBP and DAPI in response to TBI and Sodium pyruvate. There is an increase in the MBP immunostaining in the post-injury peri-lesional cortex in mTBI + vehicle group (B) compared to the uninjured cortical sections from naïve + vehicle group (A). The MBP staining is characteristically found in the cellular structures as well as fiber structures in the peri-lesional cortex, unlike the typical strand-like staining appearance (A). The impact injury has made a contused indentation in the immediate cortex making a deep recess in the tissue marked “inj” and sub-cortical capillaries marked as “c” in image B (mTBI + vehicle group). Image C is obtained from rat brain mTBI + pyruvate group. The glial limitans (GL) are breached and replaced by dense cellular layer and the presence of well-rounded capillary spaces (“c”) seen in Images B & C, shows the contused brain regions. MBP immunoreactive cells and fibers are conspicuously less in the treated brain sections compared to untreated brain sections.

#### Tau expression in TBI

Figure 6 shows the IR of Tau in the perilesional cortex as well as hippocampal regions of rat brains from mTBI (Fig. 6A) and mTBI + pyruvate group (Fig. 6B). The peri-contusional neo-cortex and adjacent fimbria/fornix of the injury show more punctate spots of Tau immunolocalization in axonal bundles. A subset of them is colocalized with the GFAP suggesting intra-cytoplasmic accumulation of Tau in astrocytes (arrows in Fig. 6A). The punctate Tau^+^ IR suggests the intra-axonal and cytoplasmic accumulation of proteolytic products of Tau suggesting active cell degeneration. The Tau^+^ IR in our study suggests that the pyruvate treatment has an increased amount of reactive gliosis occurring in the perilesional regions of the injured brain. Tau proteins are structural microtubule-binding proteins primarily localized in the axonal compartment of neurons.

#### MBP expression in MTBI

MBP originates from oligodendroglial cells and binds with myelin. As shown in Figure 7, there is a large increase in the MBP immunostaining in the post-injury peri-lesional cortex compared to the uninjured cortical sections. The MBP staining is characteristically found in the cellular structures as well as fiber structures in the peri-lesional cortex, unlike the typical strand-like staining appearance (see Fig. 7A). This might also suggest increased expression of MBP in the immediate peri-lesional cortex. Compared to the mTBI group perilesional cortex, the mTBI + pyruvate group has lesser immunoreactivity (as seen in Fig. 7C). This data suggests that the recruitment of MBP+ oligodendrocytes to the peri-lesional cortex is lesser with the pyruvate treatment compared with mTBI.

#### Nitro-tyrosine expression in mTBI

**Figure 8.**
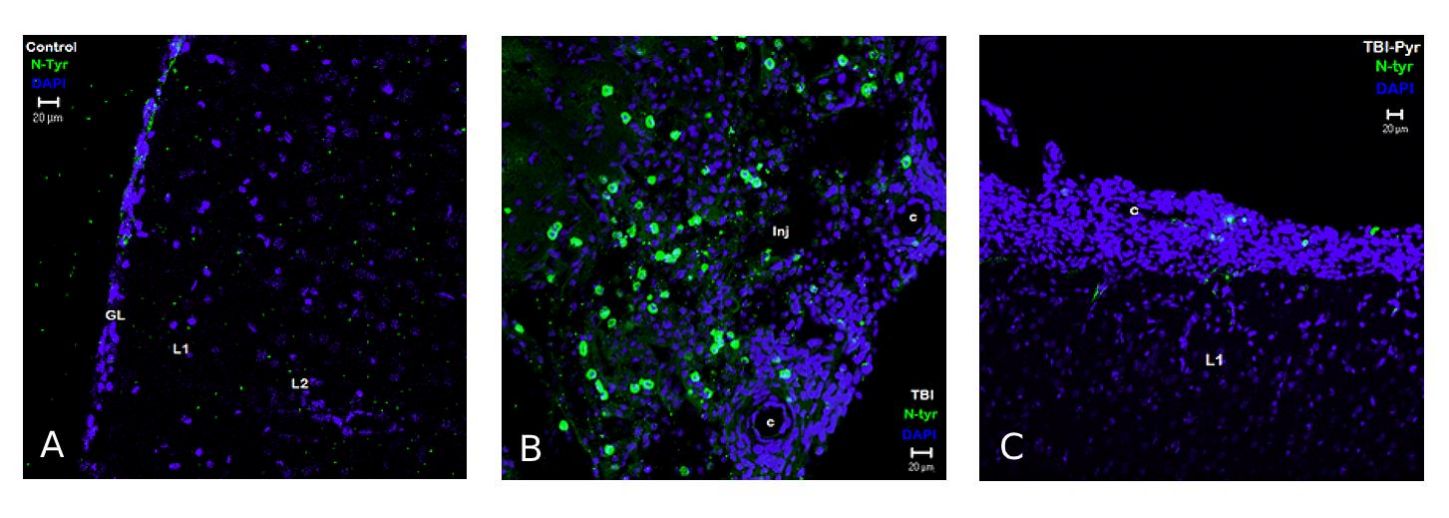
Immuno-colocalization of Nitro-tyrosine and DAPI in response to TBI and Sodium pyruvate. Nitro-tyrosine (N-Tyr) Immunoreactivity in cortical lesional areas of rat brain sections of naïve + vehicle group (A), mTBI + vehicle group (B) and mTBI + pyruvate group (C). Details of the antibody and acquisition of images are given in the method section. The iso-cortical pyramidal layers 1 and 2 are seen in the field (depicted in the picture as L1 and L2) and the ensheathing glial limitans (dense DAPI nuclear-stained layer marked as “GL” in the picture), an astroglial cell layer adjacently beneath the pia-mater. In the uninjured control cortical sections, the GL is intact, as well as L1 and L2. The N-Tyr immunoreactivity was absent in injured control sections. There was a large increase in scattered density of N-Tyr immunoreactive cells in the peri-lesional cortical sections (marked as “Inj” in the image) after one-week post-injury (“TBI”). Note that instead of GL, there is a large increase in cell density in the peri-lesional areas associated with blood capillaries (c) depicting the gliotic changes in the injured sections. In sodium pyruvate (image marked as “TBI-Pyr”) treated rat brain sections, the presence of N-Tyr immunoreactivity was found to be very less.

As shown in Figure 8, there is an increase in N-Tyr immunoreactive cells in the post-injury perilesional cortex as compared to uninjured cortical regions. In Figure 8B, the expression of N-Tyr can be seen more in the contused cortical regions, where increased cell density seen in places were glial limitans are absent and increased amounts of capillaries (“c”), rather than away from it. However, in sodium pyruvate treated animals, the expression of N-Tyr was very less in the perilesional contused regions of the cortex (Fig. 8C).

## DISCUSSION

In this study, the neuroprotective role of sodium pyruvate administration in acute mTBI was examined for the mitochondrial damage, gliotic, degenerative changes, and oxidative stress to compare against neurobehavioral and motor and sensory functional derangement. There is an overall impairment of neurobehavioral and motor functional responses, along with the decreased expression of many mitochondrial ETC enzymes, increased gliosis, neurodegenerative changes with oxidative stress, in the injured motor cortical regions, as shown by the expression of GFAP along with Tau colocalization. Oral treatment with pyruvate during the first 7 days after mTBI resulted in worsening of the behavioral and motor responses and a further increase in gliosis and neurodegeneration along with decreased myelin repair and oxidative stress, as shown by the expression of MBP and N-Tyrosine in the perilesional cortex.

### Behavioral and functional effects of mTBI and pyruvate treatment

Cognitive deficits are common causes of distress and disability after mild and moderate TBI. While there are several deficits in cognitive performances, many of these are related to learning and memory, which play an important role in the development of neurodegenerative disorders over a prolonged time.[23, 24]. In this study, we measured open field activity was measured on days 3 and 5 post mTBI to measure the general health and depressive and anxiety-like behavior. NSS-R measurement was conducted at days 3 and 5 post mTBI for measurement of overall sensory and motor functioning and the Rotarod performance of the animals was assessed at days 4 and 6 following mTBI to determine any abnormality of the motor coordination. Pyruvate treatment of TBI animals significantly prolonged the rotarod performance. The significant decrease in HA and VA and worsened neurobehavioral severity scores in the mTBI + pyruvate group suggest a certain debilitating effect of pyruvate on the acute phase of TBI. These behavioral deficits may reflect the loss of motor neurons after mTBI and other sequential neuroinflammatory and neurodegeneration events associated with pyruvate treatment as explained below. The prolonged rotarod performance might be due to increased hanging time causing latency to fall from the rotor. With worsened HA, VA, and NSS-R, the latency to fall in pyruvate treated mTBI group might occur due to the enhanced rigidity of muscles reflecting a deficient function of motor neurons of the cortex. Overall, there was a consistent lack of performance in the pyruvate treated mTBI group suggesting a worsening of motor cortical function. This is also reflected in the subsequent examination of the pathological changes in the injured cortex as revealed by the immunohistochemistry studies.

### Effect of pyruvate on mitochondrial enzymes in mTBI

In this study, the components of mitochondrial Complexes I-V were differentially affected between the hippocampus and cerebral cortex, and between contralateral and ipsilateral hemispheres of mTBI. Administration of sodium pyruvate also significantly affected the mTBI-induced expression of mitochondrial Complexes I-V in these brain regions [26]. Compared with naïve controls, mTBI injury significantly decreased ETC protein expression in the ipsilateral injury site of the hippocampus and cerebral cortex but not on the contralateral side of the brain. The effects of pyruvate treatment on ETC components are mixed, either enhancing (CI, CIII, CIV of the ipsilateral hippocampus, CI and CIII of the ipsilateral cortex, and CIII and CIV of naïve cortex), or suppressing (CII and CV of the ipsilateral and contralateral hippocampus and naïve hippocampus, CIV and CV of the cerebral cortex, or no effect (CI and CIII and CIV of naïve hippocampus, CII and CV of the contralateral hippocampus, CII and CV of naïve cortex, CI, CII and CIII of contralateral cortex), suggesting variable protecting or stabilizing effect of pyruvate on dysregulated ETC protein expressions after mTBI.

The enduring cognitive deficits and abnormal histopathology associated with mTBI may also arise from damaged mitochondria, which initiate chronic metabolic dysfunctions, cell death, and neurodegenerative process over a long time after moderate to severe TBI. Mitochondrial integrity, ETC enzyme activities are crucial to fuel high metabolic demands by central nervous system (CNS) function, neuronal firing, survival, and healing of the injured brain [27, 28]. Structural and functional damage to mitochondria is an early event following TBI. Damaged mitochondria would compromise ATP production and increase free radicals release through dysregulated ETC which are lodged in the mitochondrial matrix. Dysregulation in mitochondrial respiratory enzymes and progressive decline in brain energy metabolism have been reported for several neurodegenerative diseases [29], and are proposed as a key mechanism in AD [30, 31].

Pyruvate and mitochondrial ETC play inter-mingled roles for ATP production, ROS signaling, cell survival, apoptosis and proliferation, inflammation, cognitive, and motor functions. In aerobic conditions, pyruvate enters the citric acid cycle to yield energy-rich electron carriers that help produce ATP at the ETC. Complex I and III are the major sources of mitochondrial ROS production. Inhibition of mitochondrial complex I activity by neurotoxins trichloroethylene triggered concomitant striatonigral fiber degeneration and loss of dopamine neurons in the midbrain in animals and dose-dependently induced Parkinson's disease and motor deficits in industrial workers [32]. Excessive ROS production during reverse electron transfer from complex II to I also caused ischemia-reperfusion injury [33]. While the supplement of pyruvate protected mitochondria from glutamate excitotoxicity and promoted cell survival by attenuating Ca^2+^ overload and activating autophagy [34], pyruvate may also stimulate TBI-induced proliferation of astrogliosis by rescuing proliferation of cells with impaired ETC through stimulating aspartate synthesis [35] as supporting aspartate biosynthesis is an essential function of ETC in proliferating cells [36]. Thus, pyruvate administration may have a dual role in TBI. It is noted that compared to the non-pyruvate controls, pyruvate administration reduced Complex II and V levels in naïve animals and reduced the Complex II level in the ipsilateral hippocampus but increased Complex V level in the ipsilateral hippocampus. This may indicate an auto-downregulation mechanism of Complex II and V to gate the rate of mitochondria pyruvate metabolism in the hippocampus in the event of excess pyruvate availability. This selectivity is lost for Complex V in the ipsilateral hippocampus after TBI. However, this possibility has yet to be validated.

Like shifted branched-chain amino acid metabolism between injured neurons and astrocytes [37], pyruvate metabolism may be shifted between neurons and astroglia after TBI. The interim glycolytic products lactate and pyruvate are held in equilibrium by cytosolic lactate dehydrogenase (LDH) and mitochondrial pyruvate dehydrogenase (PDH). PDH is the rate-limiting enzyme coupling cytosolic glycolysis to mitochondrial citric acid cycle and pyruvate entry rate into the mitochondrial matrix for individual oxidative phosphorylation (OXPHOS) complexes when O_2_ (as the final electron acceptor) is sufficient under normal aerobic conditions [38]. However, under anaerobic conditions such as after TBI, both PDH activity and mitochondrial pyruvate metabolism are inhibited [10, 39, 40]. Under such conditions, excessive pyruvate supply may cause lactate acidosis, astrogliosis, and neurodegeneration as implicated in this study. PDH activity is regulated by the expression, phosphorylation, and S-glutathionylation of PDHE1α1. Phosphorylation of PDH E1α1 by PDH kinase (PDK) inhibits PDH activity whereas dephosphorylation of phosphorylated PDHE1α1 by PDH phosphatase (PDP) restores PDH activity. Significantly increased PDK isoenzymes 1-4 and significantly decreased PDH activity and PDP1 and PDP2 isoenzymes were reported in TBI [40, 41]. S-glutathionylation of PDH lowers superoxide/hydrogen peroxide release from skeletal muscle mitochondria through modification of complex I and inhibition of pyruvate uptake [42].

### Pyruvate effects on GFAP, Tau, and MBP in mTBI

Historically, models of mTBI produced some degree of neuronal degeneration through the increased expression of GFAP, MBP, and Tau proteins that are observed early after injury [43] indicating the activation of a neurodegenerative mechanism following TBI.

*Glial fibrillary acidic protein (GFAP)* is a marker of astroglial activation after TBI. Astrocytes are the major cell types in supporting neurons and brain function. In response to TBI, astrocytes become reactive and proliferated to heal the lesion and to maintain the integrity of the injured brain [44]. Previous studies have shown that GFAP activation is correlated with the severity of brain injury [22]. In this study, the level of GFAP immunoreactivity (showing the reactive gliosis) was higher in the peri-lesional cortex than in the uninjured cortical region at 7day post-injury, and higher in the injured cortical regions of pyruvate-treated animals compared to that of untreated animals, indicating a promoting effect on gliosis or accelerated healing by pyruvate.

Multiple studies previously conducted on the proliferation of astrocytes after acute brain injury showed varying results [45]. Using a mature astrocyte-specific marker, aldehyde dehydrogenase 1 family member L1 (ALDH1L1), along with BrdU, one study has shown that astrocytes in the ischemic core proliferate more than those present in < 500μm outside the core within the first 7 days post-ischemic-injury and outside astrocytes are lost due to apoptosis [46]. Studies have shown that this reaction is controlled by inflammatory mTOR [47] and STAT3 [48] signaling pathways and can be identified by the specific morphology of those astrocytes [49, 50]. Those astrocytes which were immediate responders due to inflammatory reactions were found to be round/spindle-shaped. The appendages of adjacent cells were appeared to be intertwined. It appears that this was the same inflammatory astrocytic reaction seen in our study as well. However, the proliferation of astrocytes around the injured brain region may depict a niche characteristic of astrocytes [51]. As we have not seen a major change in immunoreactivity distant to the injured regions, one can infer that this astrocytic reaction might not have yet manifested till the 7^th^ day in post mTBI.

Previous studies to explore the effect of pyruvate on the astrocytic proliferation in the peri-contusional brain regions, one week after injury, were sparingly conducted. However, one study where ethyl pyruvate intraperitoneal injections were given 10 days after spinal cord injury showed a significantly lower amount of gliosis when assessed after 10 days in the peri-lesional region [52]. In that study, however, there was no significant change in gliosis 1 mm distant to the peri-contusional region. One important clue from this lies in the activation of astrocytes. In our study, we have used sodium pyruvate, not ethyl pyruvate and the effect of additional ethyl- group is not determined earlier either. In the immediate post-injury tissue, an ester form acetate, glyceryl triacetate was shown to significantly increase ATP in the peri-contusional brain region [53]. This may be important as ATP was also found to be an important activator of astrocytic proliferation via its purinergic receptors [54]. In the current study, from the morphology of the astrocytes in the peri-contusional regions, sodium pyruvate administration appears to be inducing an early inflammatory astrocytic response. Moreover, it was found to be colocalized with Tau protein as well (Fig.6A & 6B). The colocalization of GFAP and Tau in the injured regions in the mTBI+pyruvate group, it could indicate as an active cell degradation. However, from previous studies, the astroglial proliferation in the injured region during the first 3-7 days followed by apoptosis, this may be an active pruning of astrogliosis [46]. The pyruvate might be accelerating the process. Considering the morphology of astrocytes, the initial inflammatory response of the astrocytes could be associated with degradation and the Tau expression might be an indication of that degradation due to mechanical stress and/or internalization of Tau components by astrocytes, as shown previously [55, 56]. By accelerating the inflammatory astrocytic response and Tau incorporation, pyruvate treatment might be accelerating the repair process more efficiently. Considering the above possibilities, more mechanistic studies are required to understand further the role of pyruvate in long-term astroglial changes post-TBI.

*Tau proteins* are structural microtubule-binding proteins primarily localized in the axonal compartment of neurons. Recent findings of abnormal CT in TBI suggest potential diagnostic and prognostic values of Tau protein in TBI [57]. The punctate Tau IR seen in this study is consistent with the earlier studies of increased Tau expression cell processes and soma as early as 24 hours after the injury and till one week after the injury [58]. The colocalization of Tau in the GFAP cell process and the increase in expression of GFAP in post-TBI and post-treatment tissues suggest a dual nature of Tau expression in the peri-contusional areas. In the post-injury brain, there is an acute neuronal degeneration in accordance with the severity of injury [59]. Since the Tau^+^ punctuates are appeared to be sporadically present in the peri-contusional areas and within the axonal fiber bundles of the fimbria and much less colocalized with GFAP, it is an indication of an acute degenerative process rather than recruitment and presence of astrocytes adjacent to the peri-contusional areas. There is a larger increase of Tau^+^ cells and cells expressing GFAP in the brain of pyruvate-treated mTBI animals, compared to untreated mTBI animals, with the large increase in GFAP and Tau expression primarily found in the peri-contusion cortex, fiber bundles such as fornix and corpus callosum. It is not clear if the increased Tau is due to increased astrocytes recruitment or due to an increased expression of Tau.

Astrocytes (and microglia) release a myriad of cytokines and inflammatory modulators in the peri-contusional areas. One of the major signals is the Caspase-3 triggering proteolysis. Caspase-3 is the major activator of Tau and Tau phosphorylation [60, 61]. Though Tau is expressed mainly in the neurons, the proteolytic processes that lead to the expression of Tau might be independent of the expression of other neurodegenerative markers as described earlier [59]. Therefore, the increased expression of Tau^+^ punctate depicts more widespread cell injury and degenerative processes during the acute phase of mTBI. The expression of Tau peptides could also result from a secondary injury due to the effect of inflammatory mediators released by the newly recruited astrocytes and microglia in the peri-contusion areas. More studies are required to examine the expression of different Tau proteins, Aβ, and APP pathology in conjunction with astrocytes and microglial recruitment and the presence of specific inflammatory mediators.

There are six isoforms of Tau protein, translated from two splice variants differed by their number of repeat units of microtubule-binding domains by splicing exon 10. Three of them have 3 repeat domains (3R-Tau) and the other three have four units (4R-Tau) [62, 63]. In chronic traumatic encephalopathy (CTE), which is a consequence of TBI, and in AD, any/all these six isoforms can be elevated. More details of these isoforms can be found in a recent review [64]. About 85 phosphorylation sites are present in Tau or Microtubule Associated Protein Tau (MAPT). The most common are Ser/Thr residues, preceding a Proline residue [65]. During pathological conditions, Tau can be hyperphosphorylated due to the dysfunction of a prolyl isomerase (Pin1) enzyme and detach from microtubules and accumulate in the cell called Neurofibrillary Tangles (NFT). Phosphorylated Tau (p-Tau) can be in two isomeric states (*cis* or *trans*). The critical isomerizing phospho-residue was found to be Thr231. Normally the p-Thr231-Tau is in *trans* conformation. Studies conducted in TBI using specific antibodies against the *cis* p-Tau isomer showed that the *cis* p-Tau accumulates early, within hours, following TBI and not dynamically converted to *trans-* from when there is a dysfunction of Pin1 enzyme [66]. There can be many differences in Tau pathology between CTE and AD, such as Tau deposition and progression in the brain, Tau phosphorylation sites, associated axonal injury, presence of other inclusion bodies, etc. A detailed description of the difference in Tau pathology between CTE and AD has been described recently [64].

The antibody used in the study is against the N-terminal region of the Tau protein and it can detect both *cis* and *trans* isomers of Tau protein. As Figure 6B shows, there is an increase in GFAP/Tau colocalized astrocytes in the post-treatment cohort. Most probably, the immunoreactivity colocalized with the GFAP in the injured brain regions might be the *cis*- isoform of the Tau. Considering the higher amount of inflammatory astrocytes in the injured brain region, the increased colocalization might be either similar to subpial age-related Tau astrogliopathy (ARTAG) due to mechanical stress [55] and/or internalization of Tau components by astrocytes [56]. More details of these events are described in a recent review [50]. This needs to be further evaluated in subsequent TBI studies.

*Myelin basic protein (MBP)* is an essential component of myelin formation and one of the most abundant proteins in the central nervous system (CNS). Like GFAP for astrocytes, MBP is a cellular marker for oligodendrocytes. The increased MBP immunostaining in the hippocampus of TBI and even more prominent in the TBI+ pyruvate group signifies the proliferation of oligodendrocytes in these peri-lesional areas. Since the impact of injury was 6 days prior, this suggests oligodendrocytes recruitment or a compensatory response to the injured brain while a persistent decrease in MBP levels could lead to deficits in oligodendrocytes-associated neuroplasticity in the hippocampus and or to subsequent neurodegenerative process [67]. Our results are in agreement with a previous report of extensive degradation of MBP following TBI [68]. Few studies were conducted, so far, on MBP expression in post-injury. Further studies are required to characterize the MBP expression and degradation in post-injury paradigms.

*Nitro-tyrosine protein* tyrosine nitration (N-Tyr) occurs by the action of excess production of short-lived hyper-reactive oxygen free radical species (ROS) such as peroxynitrite (ONOO^-^ ), superoxide anion (O_2_^-^), and nitric oxide (NO) has been found in various neurodegenerative disorders [69]. N-Tyr IR was previously reported elevated in the immediate post-mTBI period of rat cerebral cortical as well as hippocampal regions due to the activation of the enzyme Nitric Oxide synthase (NOS) [70]. The presence of N-Tyr IR adjacent to the contused brain regions in the current study indicates the presence of ROS in those regions. In sodium pyruvate treated rat brain sections, there is a marked reduction of N-Tyr IR cells in the perilesional cortex, indicating reduced recruitment of ROS in the injured cortex after pyruvate treatment due to its anti-inflammatory mechanisms[71]. Other studies showed similar N-Tyr results and behavioral change 13-15 days post-injury in L-Arginine-treated mTBI models [72]. These results suggest that altered expression of GFAP, Tau, and MBP could be the biomarkers of the severity of neurodegeneration following mTBI and pyruvate treatment.

In conclusion, we confirmed that TBI induced significant alterations in the expression of inflammatory and neurodegenerative proteins GFAP, Tau, MBP protein, as well as in OFA and neurobehavioral sensory and motor functioning using NSS-R and Rotarod performance. Administration of pyruvate improved mitochondrial complex 1 enzyme, and peroxide radicals associated injury in the injured hippocampus and cortex area following TBI. But pyruvate was unable to improve the motor and sensory performance as evidenced by NSS-R and OFA. The neurodegenerative and inflammatory changes in the injured areas and ipsilateral hippocampal regions suggest greater cognitive and memory impairment underlying mTBI and that it could have a wider effect with pyruvate treatment. To understand this effect, further studies using an elaborate set of behavioral studies are necessary. Moreover, as this study was explored only for 7 days post mTBI, further studies are required to elucidate the long-term effect of pyruvate treatment on neurodegeneration and behavioral outcomes in TBI animals.
